# In Memoriam—G. M. Pease, M. D.

**Published:** 1892-05

**Authors:** A. S. Larkey, S. F. Rodolph

**Affiliations:** President; Secretary


### G. M. PEASE, M. D.

At a regular meeting of the Alameda County Homœopathic Medical Society, held February 12th, 1892, the following Preamble and Resolutions were unanimously adopted:

WHEREAS, in the Divine economy of nature, it has pleased God to remove from our number one of our honorary members, Giles M. Pease, M. D., therefore

*Resolved*, That in the death of Dr. Pease we have lost an efficient member, a wise counsellor, and an eminent surgeon, and the medical profession a faithful exponent of the principles of Homœopathy.

*Resolved*, That this Preamble and these Resolutions be spread in full upon the Minutes of this Society, and a copy, properly engrossed, be sent to his family, and a copy be also sent to the *California Homœopath* for publication.

A. S. LARKEY, M. D., *President.*
S. F. RODOLPH, M. D., *Secretary.*

